# Ecotoxicity and Biodegradation of Sustainable Environment-Friendly Bone-Glue-Based Adhesive Suitable for Insulation Materials

**DOI:** 10.3390/polym14112209

**Published:** 2022-05-29

**Authors:** Klára Kobetičová, Martin Böhm, Miloš Jerman, Jaroslav Dušek, Robert Černý

**Affiliations:** Department of Materials Engineering and Chemistry, Faculty of Civil Engineering, Czech Technical University in Prague, Thákurova 7, 166 29 Prague, Czech Republic; martin.bohm@fsv.cvut.cz (M.B.); milos.jerman@fsv.cvut.cz (M.J.); jaroslav.dusek@fsv.cvut.cz (J.D.); cernyr@fsv.cvut.cz (R.Č.)

**Keywords:** ecotoxicity, biodegradation, rape straw, bone glue, sodium lignosulfonate

## Abstract

Bone glue with sodium lignosulfonate is a protein-based adhesive. Their combination leads to strong binding necessary for the achievement of adhesive properties. However, biodegradation and ecotoxicity of materials composed of bone glue and sodium lignosulfonate has never been studied before. In this paper, the biodegradation potential of the mixture of bone glue, lignosulfonate and rape straw modified by water or NaOH on an agar test with aerial molds and in acute aquatic tests with mustard, yeasts, algae and crustaceans was analyzed. Epoxy resin as an ecologically unfriendly binder was used as a negative control and pure rape straw as a background. The results indicated that all samples were covered by molds, but the samples containing straw treated by NaOH showed lower biodegradability. The ecotoxicological effects varied among the applied model organisms. *Artemia salina* was not able to survive and *S. alba* could not prolong roots in the eluates of all samples (100% inhibition). Freshwater algae (*D. subspicatus*) were not significantly affected by the samples (max. 12% inhibition, max. 16% stimulation). The biomass of yeasts (*S. cerevisae*) was strongly stimulated in the presence of eluates in a comparison to control (max. 38% stimulation).

## 1. Introduction

Ecotoxicity is a property describing the effects of commercial or natural substances and products on all compartments of the environment. Many standard bioassays with plants, animals or microorganisms have been described in the past by the Organization for Economic Co-operation and Development (OECD) [1], the International Organization for Standardization (ISO) [2] and the American Society for Testing and Materials (ASTM) [3]. With the advent of the new European legislation for Registration, Evaluation and Assessment of Chemicals (REACH) [4] in the last decade, a large number of chemicals and commercial preparations have been tested, but the study of the ecotoxicity of building materials is still lagging behind [5,6,7,8]. These are materials produced from various mixtures and substances, whether they are construction or industrial wastes containing organic pollutants and metals.

Ecotoxicity and human toxicity of artificial adhesives were partly studied in the last few years, and this information is included in the data safety sheets of the products. Epoxy resins are dangers for aquatic environments, with long-lasting effects, and formaldehyde has been confirmed as a carcinogen. However, the (eco)-toxic potential of organic natural adhesives and their properties affecting additives with the additional chemicals have not been studied yet. Determining the ecotoxic potential of such construction products is therefore very important in order to prove their safety in potential degradation and biodegradation.

Adhesives prepared from natural organic materials have been used in construction since prehistoric times. Such organic materials can be divided into two types. The first type is vegetable sources (sugars, fruit liquids) and the second type is cattle or poultry (casseins, collagens, keratins, eggs) [9,10]. All these additives are organic and not toxic for the environment, contrary to the commercial synthetic adhesives based on formaldehyde, isocyanate or epoxy resins [11]. For example, formaldehyde is a confirmed carcinogen [12,13,14] that can be released from some construction materials after their application. Epoxy resins are often used in construction applications in combination with flame retardants [15,16], but their problem lies in the uncertainty of their health safety. During their production or application, their negative effects on health can be confirmed with certainty [17], but after curing in the construction industry, together with other components, they form an insoluble material and their possible ecotoxic effectiveness is therefore still unclear. Some other adhesives are thermosets. The most commercially used thermosets are based on phenol formaldehyde (PF), resorcinol formaldehyde (RF), urea formaldehyde (UF), or melamine formaldehyde (MF) [18]. Melamine-urea-formaldehyde (MUF) adhesives, which are produced by modifying MF adhesive with urea-formaldehyde adhesives, are also used [19,20]. Alternatively, polyvinyl acetate adhesives (PVAC) can be used [21,22].

Bone glue is a nontoxic protein-based adhesive made from collagen. This polymer consisting of biodegradable organic protein molecules has usually been used as an adhesive in model making, and is also used for gluing products that are desirable to reglue in the future, e.g., during restoration or for gluing musical instruments.

In the literature, some natural fibers such as kenaf, wood, hemp, coconut, cane and straw were investigated for applications in buildings [23,24]. One of the most suitable types of blades is rape straw. It consists of cellulose and hemicellulose (80%), with the remainder being elements such as C, O, H, P, K, Ca and Mg [25]. Rapeseed straw is used as food or bedding for animals or as pellets and briquettes for heating, for paper production, and in construction as building panels and thatched roofs [26,27,28]. This material is fortunately also a very ecological product, because burning biomass is released into the air as CO_2_, which is accumulated into the plant mass by photosynthesis during its growth. Biomass burning therefore has zero carbon footprint, and has very big potential for new use in green ecological concepts. 

Sodium lignosulfonate is produced as a waste in the paper industry. It can be used as a nutrient of plants, dyes for textiles, in metallurgical engineering and the oil industry, for production of porcelain or as a plasticizer in cement-based composites [29,30,31]. Sodium lignosulfonate and technical hydrolysis lignin were also used for the production of wood fiberboards [32]. These materials are able to absorb water and it should not contain heavy metals. It can be so used for some remediation and ecological applications, e.g., as a formaldehyde scavenger [33,34,35,36].

The interaction between bone glue and sodium lignosulfonate has been studied several times [37,38,39,40]. It has been found that the adhesion capacity of the bone adhesive is increased by the addition of a certain amount of sodium lignosulfonate. Through covalent bonds between bone adhesive proteins, a dense network is formed that improves the adhesive properties [37,38,39,40].

For these reasons, bone glue combined with sodium lignosulfonate and rape straw modified by addition of H_2_O or NaOH was tested as a new natural biomaterial on an ecotoxicity (mustard test, yeast test, algal test, crustacean test) and biodegradation bioassays with molds. Epoxy resin as a presumed ecologically unfriendly adhesive instead of bone glue in samples was used as a negative control, and pure rape straw was used as a background.

## 2. Materials and Methods

Bone glue was purchased from Baltech. Ltd. (Prague, Czech Republic), sodium lignosulfonate from Stachema, Ltd. (Mělník, Czech Republic), NaOH from Sigma Aldrich. Ltd. (Prague, Czech Republic), epoxy resin (named One Resin) from Gougeon Brothers, Inc., (West Palm Beach, FL, USA). Their compositions according to the producers are shown in Table 1.

Rapeseed straw was grown in Polepy, a village with 1300 inhabitants located near Czech Central Mountains. The harvested straw was delivered to the laboratory in a plastic bag. A representative sample of straw was treated in two ways: soaking in water at 70 °C for 30 min and soaking in 2% NaOH at 25 °C. Untreated straw was used as a reference sample. The bone glue was first mixed with water in a 1:1 ratio to swell. This phase lasted 2 h. Then, the mixture of bone glue and water was heated to 70 °C, and after five minutes five percent sodium lignosulfonate was added. The mixture was maintained at 70 °C for another 2 min with stirring. The adhesive obtained was mixed with straw and pressed in a mold at a pressure of 4 MPa. The plates were pressed for 2 h.

The epoxy resin was mixed with hardener in a ratio of 2:1, then the rapeseed straw was added; the amount of epoxy material was 5% of the total weight of the mixture [40].

**Figure 1:**
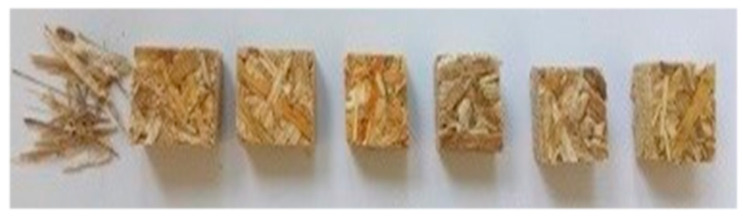
The samples (from left to right): rape straw, epoxy sample, epoxy-H_2_O sample, epoxy-NaOH sample, glue-LS sample, glue-LS-H_2_O sample, glue-LS-NaOH sample.

An example of produced samples is shown in a Figure 1.

Ultrapure deionized water (resistivity at 25 °C > 18.2 MΩ·cm) was used as the blank sample, and to obtain the leachate. The quantity of leached metals was determined using inductively coupled plasma optical emission spectrometer Agilent 5110 SVDV (ICP-OES, GenTech Scientific, Arcade, New York, NY, USA). The device was equipped with a SeaSpray glass concentric nebulizer and Autosampler SPS 4 (Agilent Technologies, Arcade, New York, NY, USA). The general settings of the device were as follows: radio frequency power 1.2 kW; sample uptake delay 18 s; rinse time 18 s; peristaltic pump rate 80 rpm. Pure argon was used for the measurement (99.996%, Linde Gas, Prague, Czech Republic) and the measurement conditions were as follows: three replicates, stabilization time 15 s, replicate read time 10 s, peristaltic pump rate 12 rpm, plasma gas flow 12 L·min^−1^, nebulizer flow 0.7 L·min^−1^, auxiliary argon flow 1 L·min^−1^. The limits of quantification (LoQs) for each analyte were determined as ten times the relative standard deviation. ICP Expert Software v. 7.4 (Agilent Technologies, Arcade, New York, NY, USA) was used for the evaluation. 

The leachate from solid samples was obtained according to the ČSN EN 12457-4 [41] standard. At the preparation of the leachate, the tested material was mixed with distilled water at a ratio of 1:10 (solid to liquid ratio). A total of 100 g of material and 1 L of distilled water was used. The prepared mixture was stirred for 24 h in overhead shaker Reax 20/4 (Heidolph Instruments, Schwabach, Germany). The leachate was filtered through filter paper (Whatman, grade 6) and analyzed. The eluates were used for the preparation of tested media in ecotoxicological bioassays or chemical analysis and their pH was measured by a PC 70 + DHS multimeter.

Yeasts *Saccharomyces cerevisiae* RIBM BP11 were donated by VÚPS, Ltd., Prague, Czech Republic. They were tested according to [42].

Eggs of *Artemia salina* were purchased from EasyFish, Ltd. (Kyjov, Czech Republic). Ten fresh-born crustaceans were placed into a control medium (30 g NaCl·L^−1^) or 100% extract (30 g NaCl·L^−1^) to a volume of 5 mL (microplate). The media were aerated for 24 h before the start of the test. The test lasted 48 h. The monitored parameter was the mortality and immobilization of crustaceans, which was evaluated according to the rules specified in the guideline after 24 and 48 h [43]. Two replicates with ten animals were used for the samples and control.

*Sinapis alba* seeds were purchased from Osiva-semena, Ltd. (Prague, Czech Republic). The seeds were pregerminated and then placed in a glass Petri dish on 15 seeds on moistened filter paper. In the case of control, distilled water was used to moisten the paper, in the case of samples their 100% leachate. The plates were covered with lids and left in an incubator at room temperature (20 ± 2) °C and in a dark place for 96 h. Root lengths of individual seeds in each dish were then measured with a ruler. Three replicates were used for the samples and the control.

Freshwater algae *Desmodesmus subspicatus* were purchased from the Institute of Botany of CR (CCALA, Ltd., Czech Academy of Science, Třeboň, Czech Republic). BB medium (CCALA, Ltd., Czech Academy of Science, Třeboň, Czech Republic) was used for algae cultivation. The test was performed according to [42].

Unspecific aerial mold community was used in the biodegradation experiment [42]. At the end of the incubation period, the growth of mold mycelium was analyzed visually under stereomicroscope and the results were evaluated according to the resistance-degree scale, with 0 indicating no growth and 5 indicating heavy mold growth [44].

The growth data were evaluated using the one-way analysis of variance (ANOVA), by means of the GraphPad InStat software (InStat version 3, San Diego, CA, USA). The multiple-comparison Dunnett test was performed at 0.05 significance level. The biodegradation data were evaluated using the one-way analysis of variance (ANOVA), by means of the GraphPad InStat software as well. The multiple-comparison Tukey–Kramer test was performed at 0.05 significance level. 

## 3. Results and Discussion

### 3.1. Chemical Analyses and pH Values

Distilled water and rape straw were measured as a background. The distilled water was without a presence of heavy metals or organic pollutants according to our previous results. The aquatic eluates of tested samples contained Al, B, Ba, Ca, Cd, Cr, Fe, K, Mg, Mn, Na, Ni, P, Si, V and Zn. The amounts of As, Cd, Cr, Ni, Pb, Hg and V were under the limit of detection in all the measured samples. The main biogenous elements C, N, O and H could not be measured because of their presence in the surrounding atmosphere.

The rape straw contained aluminum (Table 2), in addition to biogenous elements (C, O, P, N, Ca, Fe, K, Mg, Na, S and P). The negligible detected values of other elements could originate in the background of the working environment.

Sodium-lignosulphonate (LS) is composed of sodium salts C14-16-alkanhydroxy and C14-16- alkene sulfonic acids, sodium hydroxide (NaOH) and 2-oktyl-2H-isothiazol-3-on according to the producer (Stachema, Ltd., Mělník, Czech Republic). The used wood could have once been impregnated with a product containing zinc, aluminum, etc. in the past.

Glue (G) is an organic biomaterial containing a mixture of glutin and its fission products. It did not contain any heavy metals or organic pollutants according to the producer. The main component of bones is the mineral calcium phosphate, which is composed of a structure very similar to the apatite group minerals that occur naturally in Earth’s crust [45]. Determining the exact composition and crystal structure of bones is very difficult, so the following elemental composition is often used: Ca, Na, Mg, P, O, H, C, N, F, Zn [46]. In our samples, non-negligible amounts of Al, Ba and B were detected (Table 2). B is a micronutrient occurring in plants as well as animal bodies, and this is a more probable explanation for its detection in bone glue than bioaccumulation and food chain. Ba and Al are toxic elements; their occurrence may be related to industrial glue production and impurities from the production equipment. 

Epoxide (E) is a mixture of organic components (4,4’-Isopropylidenediphenol, oligomeric reaction products with 1-chloro-2,3-epoxypropane, oxirane, mono[(C12-14-alkyloxy)methyl] derivates, 4-hyroxymethyl-1,3-dioxolan-2-one, benzyl alcohol, benzoic acid, 4[{(methylphenylamino) methylene} amino]-, ethyl ester). We can suppose that it contains such elements as C, N, O, H and Cl according to the producer (Gougeon Brothers, Inc, West Palm Beach, FL, USA). The measured samples contained various levels of all analyzed elements (see Table 2). However, the elements from eluates of epoxy samples were present in lower amounts than from eluates of glue samples, generally. In addition, rape straw treated by NaOH contained similar or lower amounts of elements than the rape straw treated by water or without treatment. We can suppose that epoxy is crosslinked and traps more elements than glue. The ability of the individual elements leaching depends, apparently, on their levels in the samples as well as their composition. 

The pH values of the studied samples (except for those containing straw treated by NaOH, (see Table 3) were in a range of optimal values for freshwater organisms. *A. salina* is an organism living in brackish waters with high levels of salts and the pH values were so suitable for its life. 

### 3.2. Ecotoxicity

Ecotoxicological results of the study are presented in Supplements No. 1–4 and in Table 4. The eluate of rape straw caused 16–21% inhibition for *A. salina*, but it had a stimulate effect on the other model organisms. Rape’s eluate did not contain heavy metals, only basic nutrients (see Table 2). In the case of samples with glue and epoxy resins, a total lethality was observed for the aquatic crustacean *A. salina*. The animals were not able to hatch from the eggs for the glue, glue-LS-H_2_O, epoxy and epoxy-H_2_O samples. They had been able to be born in eluates from the samples containing NaOH, but they also died during the next day. These results could be affected by lower levels of some elements in the eluates of the glue-LS-NaOH and epoxy-NaOH samples. Another explanation could be a negative effect of gluing on the animals regardless of the adhesive mixture used. The effects of epoxy resins on crustaceans observed in this paper are in a general accordance with some other studies. Vermeirssen et al. [47] reported toxic lethal effects of epoxy paintings containing bisphenol A used on steel constructions for daphnids. Pereira et al. [48] described the effects of bisphenol A on the metabolism of proteins in daphnids. However, the effects of epoxy resins or bisphenol A on the *Artemia salina* species have never been studied before.

The seeds of *S. alba* did not prolong roots in the present study. The 100% inhibition of root prolongation could though not be explained satisfactorily. Therefore, the analyses on the subcellular level should be conducted in the future studies. The effects of various heavy metals or excess nutrients on the stress reaction of plants and the effect on their germination, growth and other metabolic processes have been reported many times before, e.g., [49,50,51,52]. On the other hand, there was also found self-production of Bisphenol A by mustard plants (*S. alba*) up to a concentration of around 8 mg/kg. In all probability BPF is a reaction product from the breakdown of the glucosinalbin with 4-hydroxybenzyl alcohol as an important intermediate [53]. The other plant species were variably sensitive to epoxy resins [54,55,56,57,58,59].

Green algae did not cause a significant sensitivity (up to 12% only) to the tested samples with glue or epoxy resins. This was not in contradiction with the results obtained by other investigators; the algae exposed to epoxy resins showed different sensitivity from nontoxicity to some metabolic effects to the decrease in their biomass [60,61,62].

The yeasts apparently profited from the substances contained in the tested samples and toxicity was not observed. The potential of yeasts *S. cerevisae* and some microbial strains (*Lactococcus lactis, Bacillus subtilis, Lactobacillus plantarum, Enterococcus faecalis*) for biodegradation of bisphenol A and the by-products of epoxy resins was observed in a study of [63]. This indicated the potential use of these organic substances as a source of nutrients corresponding to the results of this study.

### 3.3. Biodegradation Tests with Molds

**Figure 2:**
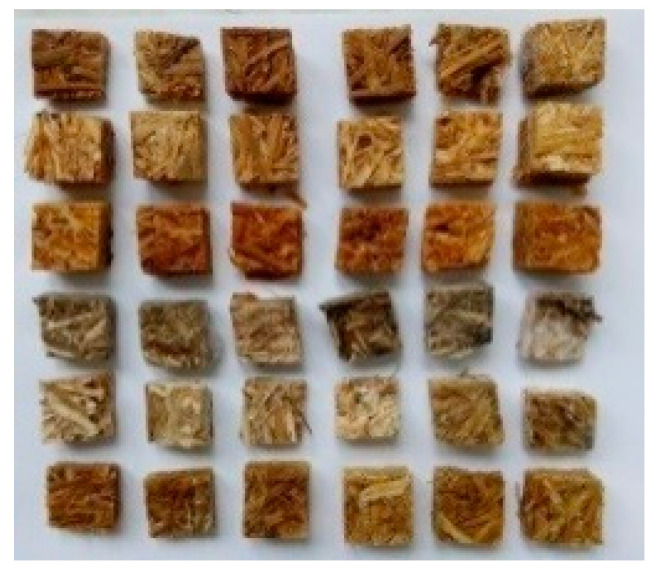
The photo of the samples covered by molds after finishing of the test (from top to bottom): glue sample, glue-H_2_O sample, glue-NaOH sample, epoxy sample, epoxy-H_2_O sample, epoxy-NaOH sample. The first three samples (from top to bottom) were sterile and the other three samples were nonsterile in each line. All samples contained straw.

**Figure 3:**
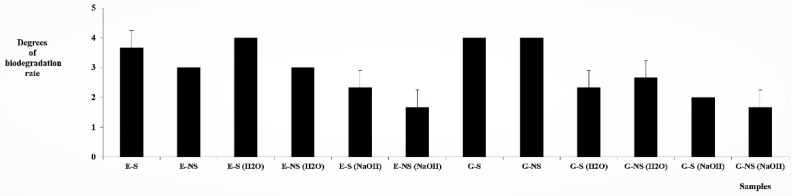
The samples covered by molds after finishing of the test (from left to right) according to degrees in [44]: E = epoxy sample, E-(H_2_O) = epoxy-straw-H_2_O sample, E-(NaOH) = epoxy-NaOH sample, G-glue sample, G-(H_2_O) = glue-H_2_O sample, G-(NaOH) = glue-NaOH sample. “S” = sterile sample, “NS” = nonsterile sample.

The microscopic observation of samples from the biodegradation tests with molds indicates that all samples were covered by molds (see Figure 2). A 6-digit scale was used to evaluate mold coverage, in which grade 0 means that the samples are not molded at all; grade 1 indicates coverage in the range of 1–25%; grade 2 in the range of 26–50%; grade 3 from 51 to 75%; grade 4 from 76 to 99%; and grade 5 = 100% sample coverage. This method of evaluation is often preferred in mold, because mold cover is much worse in depth than biodegradation by wood-destroying fungi [64]. Therefore, the samples were examined under a stereo microscope, and according to the percentage of their surface coverage they were divided into individual stages (see Figure 3). The best results (the least bio-attacked samples) were found for the glue samples and the epoxy samples, both treated by NaOH (up to 50%). The remaining samples were covered by molds in a range from 50 to 100 %. The most damaged samples were pure glue and epoxy-resin samples without treatment and epoxy samples treated by water (see Figure 2 and Figure 3). The possible attack of epoxide by molds was also described by Bae et al. [65], where the authors confirmed that epoxy-cured containers can promote the growth of microorganisms more than stainless steel.

Statistically significant differences (Tukey–Kramer test, one-way ANOVA) were found for E-S vs. E-NS (NaOH), E-S vs. G-S-LS (NaOH), E-S vs. G–LS-NS (NaOH) at the *** *p* < 0.001 level, E-S vs. E-S (NaOH) and E-S vs. G-LS-S (H_2_O) at the ** *p* < 0.01 level, E-S vs. G-LS-NS (H_2_O) and E-NS vs. E-NS (NaOH) * *p* < 0.05 level (see Supplement No. 5 and No. 6). The probability and number of stars indicate the relevance of statistical significance. Pure epoxide samples demonstrated the highest significance compared to the glue sample. Sterilization by UV lamp had an apparent effect on the occurrence of molds in the case of epoxide samples with NaOH treatment. Lower differences were found between glue and epoxide samples without a connection to sterilization.

The biological degradation of the samples analyzed in this paper has not been studied yet and for this reason we were not able to compare the data with other investigators. In the present study, humidity around samples was high thanks to the covered Petri glass containing moistened agar (3%). Mold molting was thus anticipated and unavoidable. The sterilization of samples before the start of the test did not have any effect on the biodegradation for glue samples. The sterile samples with epoxy resins were surprisingly more covered by molds than samples without sterilization, but these discrepancies were not confirmed statistically. 

It is apparent that organic materials such as rape straw, lignosulphonate or glue are not able to resist to biological attacks. The results from the previous studies indicated that straw treated by NaOH had the highest matrix density whether glue or epoxy was used as the adhesive [40]. In addition, rape straw treated by water had the highest porosity but the humidity was highest for the straw with NaOH. For the composite materials containing rape straw without treatment and with water treatment, the swelling was several times lower. The authors of [40] thus concluded that the rape straw from their study can be used effectively only up to the air relative humidity of 75% and is suitable for dry environments only, such as cladding and insulation in construction, or for packaging purposes.

## 4. Conclusions

Bone glue in combination with sodium lignosulphonate was found applicable for the production of a natural adhesive in insulation materials based on rape straw. The materials containing natural bone glue were similarly toxic to characteristic organisms (invertebrates and higher plants) as those with epoxy resins, even if the rape straws were treated by water or NaOH before the sample preparation. The treatment of rape straw by NaOH for insulative purposes seems to be the most interesting from the ecological point of view. The application of NaOH on straw in both glue and epoxide samples led to somewhat lower toxicity than the application of water for algae, and also partly for artemia (after 24 h exposition).

The ecotoxicological results indicated different ecotoxic potential for various organisms. Toxicity was found for higher plants and for invertebrates; stimulation was observed for microorganisms (algae, yeasts and molds in biodegradation tests). However, some other ecotoxicological tests with soil or aquatic organisms should be performed with materials based on bone glue in the future, because the presented results indicated a possible toxic potential of bone glue itself, although not higher than epoxide resin.

The presented results indicated that samples were covered by molds independently on the used natural (bone glue) or artificial (epoxide) components. Apparently, the NaOH application on rape straw followed by swelling led to lower bioavailability of water [64] for molds, and their lower occurrence on the cover of samples in comparison to untreated straw or straw treated by water. Nevertheless, some follow-up research of biodegradability should be conducted, including long-lasting experiments with lower humidity of the environment, experiments based on artificial weathering or experiments performed in real outdoor/indoor conditions.

## Figures and Tables

**Table 1:** Chemical composition of used materials.

Chemical	Composition
Bone glue	glutin and its fission products
Sodium lignosulfonate	sodium salts C14-16-alkanhydroxy, C14-16- alkene sulfonic acids, sodium hydroxide, 2-oktyl-2H-isothiazol-3-on
Epoxy resin	4,4’-Isopropylidenediphenol, oxirane, mono[(C12-14-alkyloxy)methyl] derivates, 4-hyroxymethyl-1,3-dioxolan-2-one, benzyl alcohol, benzoic acid, 4[{(methylphenylamino) methylene} amino]-, ethyl ester

**Table 2:** Chemical analysis of the tested samples—their eluates (in ppm).

Sample	Al	B	Ba	Ca	Fe	K	Mg	Mn	Na	P	Si	Zn
Dist. water	0.00	0.00	0.00	0.00	0.00	0.00	0.00	0.00	0.00	0.00	0.00	0.00
Rape straw (pure)	0.00	0.00	0.00	32.2	0.03	41.7	4.00	0.10	5.21	0.00	0.20	0.02
Sulfonate (pure)	0.00	0.00	0.00	1.50	0.01	0.06	0.20	0.00	1.44	0.00	0.10	0.01
Glue (pure)	0.09	1.00	0.20	191	0.44	>325	41.2	0.82	>103	1.40	0.30	2.98
Glue-LS-NaOH	0.20	1.00	0.03	16.6	0.35	234	23.7	0.08	>22	0.60	0.70	1.06
Glue-LS-H_2_O	0.13	0.70	0.06	127	0.52	227	24.3	0.34	>66	0.40	2.60	2.51
Epoxide (pure)	0.02	1.40	0.15	186	0.11	288	35.5	0.71	87.9	2.30	0.00	0.17
Epoxide-NaOH	0.07	1.00	0.04	14.8	0.20	149	15.7	0.11	>27	0.90	0.30	0.11
Epoxide-H_2_O	0.03	0.60	0.06	102	0.08	91.8	15.2	0.21	48.3	0.50	1.40	0.10

**Table 3:** pH values of the tested samples (the eluates).

Sample	pH Value
Distilled water	6.9
Rape straw (pure)	6.5
Glue (pure)	6.5
Glue-LS-NaOH	9.5
Glue-LS-H_2_O	6.9
Epoxide (pure)	6.0
Epoxide-NaOH	9.5
Epoxide-H_2_O	7.6

**Table 4:** Summary results of the ecotoxicological tests (the inhibitions expressed in %tiles in comparison to controls).

			Inhibition	(%)	
Sample	Yeasts	Artemia	Artemia	Mustard	Algae
	24 h	24 h	48 h	96 h	72 h
	Growth Rate	Mortality	Mortality	Root Elongation	Growth Rate
Control	0	0	0	0	0
Rape straw (pure)	−82	16	21	−6	−26
Glue (pure)	−89	100	100	100	12
Glue-LS-straw + NaOH	−34	74	100	100	0
Glue-LS-straw + H_2_O	−21	100	100	100	10
Epoxy (pure)	−100	100	100	100	0
Epoxy-straw + NaOH	−37	5	100	100	−16
Epoxy-straw + H_2_O	−38	100	100	100	12

## Data Availability

All data are included in the paper or in the supplementary data.

## Supplementary Materials

The following supporting information can be downloaded at: https://www.mdpi.com/article/10.3390/polym14112209/s1, Table S1: Ecotoxicological data—algae; Table S2: Ecotoxicological data—yeast; Table S3: Ecotoxicological data—crustacean; Table S4: Ecotoxicological data—mustard, Table S5: Average resistance rating for the tested samples; Table S6: Statistical analysis of biodegradation test among the tested samples.

## References

[1] OECD. https://www.oecd.org/.

[2] ISO. https://www.iso.org/home.html.

[3] ASTM. https://www.astm.org/.

[4] REACH. https://europa.eu/youreurope/business/product-requirements/chemicals/registering-chemicals-reach/index_cs.htm.

[5] Pacheco-Torgal F., Jalali S. (2019). Toxicity of building materials: A key issue in sustainable construction. Int. J. Sustain. Eng..

[6] Krüger O., Kalbe U., Richter E., Egeler P., Römbke J., Berger W. (2013). New approach to the ecotoxicological risk assessment of artificial outdoor sporting grounds. Environ. Pollut..

[7] Baderna D., Lomazzi E., Passoni A., Pogliaghi A., Petoumenou M.I., Bagnati R., Lodia M., Viarengo A., Sforzini S., Benfenati E. (2015). Chemical characterization and ecotoxicity of three soil foaming agents used in mechanized tunnelling. J. Hazard. Mater..

[8] Gartiser S., Heisterkamp I., Schoknecht U., Bandow N., Burkhardt N.M., Ratte M., Ilvonen O. (2017). Recommendation for a test battery for the ecotoxicological evaluation of the environmental safety of construction products. Chemosphere.

[9] Sickels L.B. (1980). Organic additives in mortars. Edinb. Univ. Res. J. Architect..

[10] Loaiza A., Garcia E., Colorado E.A. (2019). Evaluation of asphalt binder blended with coconut coir dust and residual coconut fibers for structural applications. Rev. Constr..

[11] Lee J.H., Kim J., Kim S., Kim J.T. (2013). Characteristics of Particleboards Using Tannin Resin as Novel Environment-Friendly Adhesion System. Indoor Built Environ..

[12] Protano C., Buomprisco G., Cammalleri V., Poceni R.N., Marotta D., Simonazzi S., Cardoni F., Petyx M., Iavicoli S., Vitali M. (2021). The Carcinogenic Effects of Formaldehyde Occupational Exposure: A Systematic Review. Cancers.

[13] Wi S., Park J.H., Kim J.U., Kim S. (2021). Evaluation of environmental impact on the formaldehyde emission and flame-retardant performance of thermal insulation materials. J. Hazard. Mater..

[14] Kristak S., Antov P., Bekhta P., Lubis M.A.R., Heri I.A., Réh R., Sedliacik J., Savov V., Taghiyari H.R., Papadopoulos A.N. (2022). Recent Progress in Ultra-Low Formaldehyde Emitting Adhesive Systems and Formaldehyde Scavengers in Wood-Based Panels: A Review. Wood Mater. Sci. Eng..

[15] Jin F.L., Li X., Park S.J. (2015). Synthesis and application of epoxy resins: A review. J. Ind. Eng. Chem..

[16] Liu Q., Wang D., Li Z., Li Z., Peng X., Liu C., Zhang Y., Zheng P. (2020). Recent Developments in the Flame-Retardant System of Epoxy Resin. Materials.

[17] ECHA. https://european-union.europa.eu/institutions-law-budget/institutions-and-bodies/institutions-and-bodies-profiles/echa_cs.

[18] Cosereanu C., Cerbu C. (2019). Rape/wood particleboard. BioResources.

[19] Mirski R., Derkowski A., Dziurka D., Wieruszewski M., Dukarska D. (2020). Effects of chip type on the properties of chip-sawdust boards glued with polymeric diphenyl methane diisocyanate. Materials.

[20] Mantanis G.I., Athanassiadou E.T., Barbu M.C., Wijnendaele K. (2017). Adhesive systems used in the European particleboard, MDF and OSB industries. Wood Mater. Sci. Eng..

[21] Sunardi M., Fawaid R., Lusiani S.B., Kesworo A., Widodo T.D. (2019). The Effect of Wood Sawdust Mesh Combination on Mechanical Behaviour of Particle Board. IOP Conf. Ser. Mater. Sci. Eng..

[22] Budakci M. (2010). The determination of adhesion strength of wood veneer and synthetic resin panel (laminate) adhesives. Wood Res..

[23] Berardi U., Iannace G. (2015). Acoustic characterization of natural fibers for sound absorption applications. Energy Build..

[24] Berardi U., Iannace G. (2017). Predicting the sound absorption of natural materials: Best fit inverse laws for the acoustic impedance and the propagation constant. Appl. Acoust..

[25] Richter R., Římovský K. (1995). Organická Hnojiva, Jejich Výroba a Použití.

[26] Strehler A. (1994). Aufbereitung und Verfeuerung von Biomase als Festbrennstoff. Energie aus Biomase.

[27] Rahim M., Douzane O., Tran Le A.D., Promis G., Langlet T. (2016). Characterization and comparison of hygric properties of rape straw concrete and hemp concrete. Constr. Build. Mater..

[28] Ahmad M.R., Chen B. (2020). Influence of type of binder and size of plant aggregate on the hygrothermal properties of bio-concrete. Constr. Build. Mater..

[29] Zhang M.H., Sisomphon K., Ng T.S., Sun D.J. (2010). Effect of superplasticizers on workability retention and initial setting time of cement pastes. Constr. Build. Mater..

[30] Tantawi S.H., Selim I.Z. (2004). Role of some concrete admixtures on the resistivity of cement pastes and reinforced steel. Bull. Electrochem..

[31] Topcu I.B., Atesin O. (2016). Effect of high dosage lignosulphonate and naphthalene sulphonate based plasticizer usage on micro concrete properties. Constr. Build. Mater..

[32] Guo M.H., Wang Y., Liu F. (2010). Performance Analysis of Ammonium Lignosulfonate/Urea Formaldehyde-free Fiberboards. Adv. Mater. Res..

[33] Chupin L., Charrier B., Pizzi A., Perdomo A., Bouhtoury C.E. (2015). Study of thermal durability properties of tannin–lignosulfonate adhesives. J. Therm. Anal. Calorim..

[34] Antov P., Savov V., Trichkov N., Krišťák L., Réh R., Papadopoulos A.N., Takhiyari H.R., Pizzi A., Kunecová D., Pachikova M. (2021). Properties of High-Density Fiberboard Bonded with Urea–Formaldehyde Resin and Ammonium Lignosulfonate as a Bio-Based Additive. Polymers.

[35] Hemmila V., Adamopoulos S., Hosseinpourpia R., Ahmed S.A. (2019). Ammonium Lignosulfonate Adhesives for Particleboards with pMDI and Furfuryl Alcohol as Crosslinkers. Polymers.

[36] Antov P., Savov V., Mantanis G.I., Neykov N. (2020). Medium-density fibreboards bonded with phenol-formaldehyde resin and calcium lignosulfonate as an eco-friendly additive. Wood Mater. Sci. Eng..

[37] Nguyen D.M., Grillet A.C., Diep T.M.H., Ha T.C.N., Woloszyn M. (2017). Hygrothermal properties of bio-insulation building materials based on bamboo fibers and bio-glues. Constr. Build. Mater..

[38] Nguyen D.M., Grillet A.C., Bui Q.B., Diep T.M.H., Woloszyn M. (2018). Building bio-insulation materials based on bamboo powder and bio-binders. Constr. Build. Mater..

[39] Kunanopparat T., Menut P., Morel M.H., Guilbert S. (2012). Improving wheat gluten materials properties by kraft lignin addition. J. Appl. Polym. Sci..

[40] Dušek J., Jerman M., Podlena M., Böhm M., Černý R. (2021). Sustainable composite material based on surface-modified rape straw and environment-friendly adhesive. Constr. Build. Mater..

[41] (2003). Characterisation of Waste-Leaching-Compliance Test for Leaching of Granular Waste Materials and Sludges Part 2: One Stage Batch Test at a Liquid to Solid Ratio of 10 I/kg for Materials with Particle Size Below 4 mm.

[42] Kobetičová K., Fořt J., Černý R. (2020). Interactions of superabsorbent polymers based on acrylamide substances with microorganisms occurring in human dwellings. Ecotoxicol. Environ. Saf..

[43] (2004). OECD Guidelines for the Testing of Chemicals. ^Daphnia^ sp. Acute Immobilisation Test.

[44] (1997). Plastics. Evaluation of the Action of Microorganisms.

[45] Wopenka B., Pasteris J.D. (2005). A mineralogical perspective on the apatite in bone. Mater. Sci. Eng. C.

[46] Skinner H.C.W. (2005). Biominerals. Mineral. Mag..

[47] Vermeirssen E.L.M., Dietschweiler C., Werner I., Burkhardt M. (2017). Corrosion protection products as a source of bisphenol A and toxicity to the aquatic environment. Water Res..

[48] Pereira E.O.A., Labine L.M., Kleywegt S., Jobst K.J., Simpson A.J., Simpson M.J. (2011). Metabolomics Reveals That Bisphenol Pollutants Impair Protein Synthesis-Related Pathways in *Daphnia magna*. Metabolites.

[49] Maisto G., Manzo S., De Nicola F., Carotenuto R., Rocco A., Alfani A. (2011). Assessment of the effects of Cr, Cu, Ni and Pb soil contamination by ecotoxicological tests. J. Environ. Monit..

[50] Baran A., Tarnawski M., Koniarz T., Szara M. (2019). Content of nutrients, trace elements, and ecotoxicity of sediment cores from Ro(z) over dotnow reservoir (Southern Poland). Environ. Geochem. Health.

[51] Wisniewska M., Kaminski A., Pusz A. (2019). Phytotoxicity of metal-contaminated soils. Przem. Chem..

[52] Plekhanova I.O., Zolotareva O.A., Tarasenko I.D., Yakovlev A.S. (2019). Assessment of Ecotoxicity of Soils Contaminated by Heavy Metals. Eurasian Soil Sci..

[53] Zoller O., Bruschweiler B.J., Magnin R., Reinhard H., Rhyn P., Rupp H., Zeltner S., Felleisen R. (2016). Natural occurrence of bisphenol F in mustard. Food Addit. Contam. Part A-Chem. Anal. Control Expo. Risk Assess..

[54] Nakajima N., Ohshima Y., Serizawa S., Kouda T., Edmonds J.S., Shiraishi F., Aono M., Kubo A., Tamaoki M., Saji H. (2002). Processing of bisphenol A by plant tissues: Glucosylation by cultured BY-2 cells and glucosylation/translocation by plants of *Nicotiana tabacum*. Plant Cell Physiol..

[55] Ferrara G., Loffredo E., Senesi N. (2016). Phytotoxic, clastogenic and bioaccumulation effects of the environmental endocrine disruptor bisphenol A in various crops grown hydroponically. Planta.

[56] Luo M., Gu S.H., Zhao S.H., Zhang F., Wu N.H. (2006). Rice GTPase OsRacB: Potential accessory factor in plant salt-stress signaling. Acta Biochim. Biophys. Sin..

[57] Speranza A., Crosti P., Malerba M., Stocchi O., Scoccianti V. (2011). The environmental endocrine disruptor, bisphenol A, affects germination, elicits stress response and alters steroid hormone production in kiwifruit pollen. Plant Biol..

[58] Qiu Z.Y., Wang L.H., Zhou Q. (2013). Effects of bisphenol A on growth, photosynthesis and chlorophyll fluorescence in above-ground organs of soybean seedlings. Chemosphere.

[59] Hu H., Wang L., Wang Q., Jiao L., Hua W., Zhu Q., Huang X. (2014). Photosynthesis, chlorophyll fluorescence characteristics, and chlorophyll content of soybean seedlings under combined stress of bisphenol A and cadmium. Environ. Toxicol. Chem..

[60] Nakajima N., Teramoto T., Kasai F., Sano T., Tamaoki M., Aono M., Kubo A., Kamada H., Azumi Y., Saji H. (2007). Glycosylation of bisphenol A by freshwater microalgae. Chemosphere.

[61] Duan L.Y., Chen Q., Duan S.S. (2019). Transcriptional Analysis of Chlorella pyrenoidosa Exposed to Bisphenol A. Int. J. Environ. Res. Public Health.

[62] Bell A.M., Baier R., Kocher B., Reifferscheid G., Buchinger S., Ternes T. (2020). Ecotoxicological characterization of emissions from steel coatings in contact with water. Water Res..

[63] Kyrila G., Katsoulas A., Schoretsanati V., Rigopoulos A., Rizou E., Douůgeridou S., Sarli V., Samanidon V., Touraki M. (2021). Bisphenol A removal and degradation pathways in microorganisms with probiotic properties. J. Hazard. Mater..

[64] Kwasniewska-Sip P., Cofta G., Nowak P.B. (2018). Resistance of fungal growth on Scots pine treated with caffeine. Int. Biodeter. Biodegr..

[65] Bae B., Jeong J.H., Lee S.J. (2020). The quantification and characterization of endocrine disruptor bisphenol—A leaching from epoxy resin. Water. Sci. Technol..

