# Supplementary material for: Ecotoxicity and Biodegradation of Sustainable Environment-Friendly Bone-Glue-Based Adhesive Suitable for Insulation Materials
Source: Polymers (Basel). 2022 May 29;14(11):2209. doi: 10.3390/polym14112209 (PMC9182703; doi:10.3390/polym14112209)
Supplement: Supplementary file 1 [file polymers-14-02209-s001.zip › polymers-1732732-supplementary.pdf]

Table S1. Ecotoxicological data – algae.

**Algae** *Desmodesmus subspicatus*	**0.day cells/ml**	**72 hours cells/ml**	**ln (0 time)**	**ln (72 hours)**	**growth rate**	**mean**	**SD**	**Inhibition**	**Inhibition (%)**
control	10 000	1 168 000	9.21	13.97	4.76				
control		1 023 000		13.84	4.63				
control		904000		13.71	4.50	4.63	0.13	0.00	0
rape		2 645 000		14.79	5.58				
rape		5 535 000		15.53	6.32				
rape		2 889 000		14.88	5.67	5.85	0.40	-0.26	-26.40
glue		699 000		13.46	4.25				
glue		569 700		13.25	4.04				
glue		539 000		13.20	3.99	4.09	0.14	0.12	11.63
glue-straw+NaOH		1 120 000		13.93	4.72				
glue-straw+NaOH		1 114 000		13.92	4.71				
glue-straw+NaOH		907 000		13.72	4.51	4.65	0.12	0.00	-0.34
glue-straw+H_2O		702 000		13.46	4.25				
glue-straw+H_2O		650 000		13.38	4.17				
glue-straw+H_2O		609 000		13.32	4.11	4.18	0.07	0.10	9.77
epoxy resin		1 044 000		13.86	4.65				
epoxy resin		1 043 000		13.86	4.65				
epoxy resin		1 050 000		13.86	4.65	4.65	0.00	0.00	-0.41
epoxy resin-straw+NaOH		2 133 000		14.57	5.36				
epoxy resin-straw+NaOH		1 743 000		14.37	5.16				
epoxy resin-straw+NaOH		2 490 000		14.73	5.52	5.35	0.18	-0.15	-15.46
epoxy resin -straw+H_2O		675 000		13.42	4.21				

epoxy resin-straw+H_2O	598 000	13.30	4.09				
epoxy resin-straw+H_2O	534 000	13.19	3.98	4.09	0.12	0.12	11.60

Table S2. Ecotoxicological data – yeasts.

**Yeasts** *Saccharomyces cerevisae*	**0.day cells/ml**	**24 hours cells/ml**	**ln (0.time)**	**ln (24 hours)**	**growth rate**	**mean**	**SD**	**Inhibition**	**Inhibition (%)**
control	10 000	70 000	9.210	11.16	1.95				
control		71 000		11.17	1.96				
control		71000		11.17	1.96	1.96	0.01	0.00	0
rape		340 000		12.74	3.53				
rape		352 000		12.77	3.56				
rape		369 000		12.82	3.61	3.57	0.04	-0.82	-82.33
glue		415 000		12.94	3.73				
glue		387 000		12.87	3.66				
glue		395 000		12.89	3.68	3.69	0.04	-0.89	-88.50
glue-straw+NaOH		132 000		11.79	2.58				
glue-straw+NaOH		144 000		11.88	2.67				
glue-straw+NaOH		140 000		11.85	2.64	2.63	0.04	-0.34	-34.44
glue-straw+H_2O		94 000		11.45	2.24				
glue-straw+H_2O		122 000		11.71	2.50				
glue-straw+H_2O		105 000		11.56	2.35	2.36	0.13	-0.21	-20.92

epoxy resin	480 000	13.08	3.87				
epoxy resin	509 000	13.14	3.93				
epoxy resin	475 000	13.07	3.86	3.89	0.04	-0.99	-98.80
epoxy resin-straw+NaOH	144 000	11.88	2.67				
epoxy resin-straw+NaOH	143 000	11.87	2.66				
epoxy resin-straw+NaOH	150 000	11.92	2.71	2.68	0.03	-0.37	-36.98
epoxy resin-straw + H_2O	151 000	11.93	2.71				
epoxy resin-straw + H_2O	152 000	11.93	2.72				
epoxy resin-straw + H_2O	147 000	11.90	2.69	2.71	0.02	-0.38	-38.49

Table S3. Ecotoxicological data-crustacean.

**Crustacean** *Artemia salina*	**24 hours** Number of survived animals	**24 hours Inhibition (%)**	**48 hours** Number of survived animals	**48 hours** Inhibition (%)
control	19	0	19	0
rape	16	16	15	21
glue	0	100	0	100
glue-straw+NaOH	5	74	0	100
glue-straw+H_2O	0	100	0	100
epoxy resin	0	100	0	100
epoxy resin-straw+NaOH	18	5	0	100
epoxy resin-straw + H_2O	0	100	0	100

Table S4. Ecotoxicological data - mustard.

**mustard** *Sinapis alba*				**Length of roots (mm)**														
	**mean**	**SD**	**Inhibition (%)**	**1**	**2**	**3**	**4**	**5**	**6**	**7**	**8**	**9**	**10**	**11**	**12**	**13**	**14**	**15**
control	49			66	81	43	55	97	24	70	51	53	14	49	79	45	1	1
control	28			51	28	22	22	24	41	40	5	30	25	29	34	45	23	1
control	18			41	20	20	22	25	34	44	5	8	5	8	20	22	1	1
mean	32	15.43	-															
rape	37			55	82	11	10	93	76	55	54	44	65	1	1	1	1	1
rape	29			5	29	12	88	12	15	16	82	33	66	79	1	1	1	1
rape	34			53	11	10	47	63	77	62	95	55	38	1	1	1	1	1
	33	4	-6															
glue	0	0	100	0	0	0	0	0	0	0	0	0	0	0	0	0	0	0
glue	0	0	100	0	0	0	0	0	0	0	0	0	0	0	0	0	0	0
glue	0	0	100	0	0	0	0	0	0	0	0	0	0	0	0	0	0	0
glue-straw+NaOH	0	0	100	0	0	0	0	0	0	0	0	0	0	0	0	0	0	0
glue-straw+NaOH	0	0	100	0	0	0	0	0	0	0	0	0	0	0	0	0	0	0
glue-straw+NaOH	0	0	100	0	0	0	0	0	0	0	0	0	0	0	0	0	0	0
glue-straw+H_2O	0	0	100	0	0	0	0	0	0	0	0	0	0	0	0	0	0	0
glue-straw+H_2O	0	0	100	0	0	0	0	0	0	0	0	0	0	0	0	0	0	0
glue-straw+H_2O	0	0	100	0	0	0	0	0	0	0	0	0	0	0	0	0	0	0
epoxy resin	0	0	100	0	0	0	0	0	0	0	0	0	0	0	0	0	0	0
epoxy resin	0	0	100	0	0	0	0	0	0	0	0	0	0	0	0	0	0	0
epoxy resin	0	0	100	0	0	0	0	0	0	0	0	0	0	0	0	0	0	0
epoxy resin-straw+NaOH	0	0	100	0	0	0	0	0	0	0	0	0	0	0	0	0	0	0

epoxy resin-straw+NaOH	0	0	100	0	0	0	0	0	0	0	0	0	0	0	0	0	0	0
epoxy resin-straw + NaOH	0	0	100	0	0	0	0	0	0	0	0	0	0	0	0	0	0	0
epoxy resin-straw + H_2O	0	0	100	0	0	0	0	0	0	0	0	0	0	0	0	0	0	0
epoxy resin-straw + H_2O	0	0	100	0	0	0	0	0	0	0	0	0	0	0	0	0	0	0
epoxy resin-straw + H_2O	0	0	100	0	0	0	0	0	0	0	0	0	0	0	0	0	0	0

Table S5. Average resistance rating for the tested samples. Resistance rating scale: 0 – no visible growth under the microscope, 1 – growth invisible to the naked eye, but clearly visible under the microscope, 2 – coverage of up to 25% of the tested sample, 3 – coverage of up to 50% of the tested sample, 4 – coverage of more than 50% of the tested sample, 5 – intensive growth, covering the entire surface of the tested sample. The abbreviations: E = epoxide, G = glue, S = sterile, NS = non-sterile.

Sample	Replic No. 1	Replic No. 2	Replic No. 3	Mean	SD
E-S	3	4	4	3.67	0.58
E-NS	3	3	3	3.00	0.00
E-S+H_2O	4	4	4	4.00	0.00
E-NS+H_2O	3	3	3	3.00	0.00
E-S+NaOH	2	3	2	2.33	0.58
E-NS+NaOH	2	2	1	1.67	0.58
G-S	4	4	4	4.00	0.00
G-NS	4	4	4	4.00	0.00
G-S+H_2O	3	2	2	2.33	0.58
G-NS+H_2O	2	3	3	2.67	0.58
G-S+NaOH	2	2	2	2.00	0.00
G-NS+NaOH	2	2	1	1.67	0.58

Table S6. Statistical analysis of biodegradation test among the tested samples (Tukey-Kramer test, one-way Anova).

comparing the differences between two samples	differences value	p-value

E-S vs E-NS	1.000	ns P>0.05
E-S vs E-S (H_2O)	0.000	ns P>0.05
E-S vs E-NS (H_2O)	1.000	ns P>0.05
E-S vs E-S (NaOH)	1.667	** P<0.01
E-S vs E -NS (NaOH)	2.333	*** P<0.001
E-S vs G-S	0.000	ns P>0.05
E-S vs G-NS	0.000	ns P>0.05
E-S vs G-S (H_2O)	1.667	** P<0.01
E-S vs G-NS (H_2O)	1.333	* P<0.05
E-S vs G-S (NaOH)	2.000	*** P<0.001
E-S vs G -NS (NaOH)	2.333	*** P<0.001
E-NS vs E-S (H_2O)	1.000	ns P>0.05
E-NS vs E-NS (H_2O)	0.000	ns P>0.05
E-NS vs E-S (NaOH)	0.667	ns P>0.05
E-NS vs E -NS (NaOH)	1.333	* P<0.05
E-NS vs G-S	-1.000	ns P>0.05
E-NS vs G-NS	-1.000	ns P>0.05
